# Efficacy of conversion therapy on initially unresectable locally advanced rectal cancer

**DOI:** 10.7150/jca.53824

**Published:** 2021-05-27

**Authors:** Tianyu Liu, Wenju Chang, Jian Wang, Li Ren, Ye Wei, Xian Zhang, Yijiao Chen, Wentao Tang, Mingliang Wang, Zhaochong Zeng, Jianmin Xu

**Affiliations:** 1 Colorectal Cancer Center, Department of General Surgery, Zhongshan Hospital, Fudan University, Shanghai, China.; 2 Shanghai Engineering Research Center of Colorectal Cancer Minimally Invasive Technology (17DZ2252600), Shanghai, China.; 3 Department of Radiotherapy; Zhongshan Hospital, Fudan University, Shanghai, China.; 4 Department of Radiology, Zhongshan Hospital, Fudan University, Shanghai, China.

**Keywords:** Unresectable, Rectal Cancer, Conversion Therapy, Prognosis.

## Abstract

**Background and purpose:** Research on the efficacy of conversion therapy for initially unresectable mid-low rectal cancer (IURC) remained limited. This study aimed to assess the efficacy and safety of the conversion regimen for IURC and analyze the long-term outcomes of these patients.

**Methods:** We retrospectively analyzed the data of clinically diagnosed IURC patients who received conversion therapy between October 2010 and April 2017. The conversion therapy consisted of long-term radiation, concurrent chemotherapy, delayed surgery and consolidation chemotherapy. The primary end point was the rate of R0 resection, and other short- and long-term outcomes were analyzed.

**Results:** Sixty-one patients were enrolled in this study. After conversion therapy, 51 (83.6%) patients received R0 resection. The rates of pathologic complete response and downstaging were 16.4% and 62.3%, respectively. The rate of grade 3-4 chemoradiotherapy-related toxicity events was 13.1%. The overall survival at 3 years was 75.4% in all patients, and the disease-free survival at 3 years was 72.5% in patients who received R0 resection.

**Conclusion:** The conversion regimen showed a high conversion resection rate and good survival outcomes in IURC patients, and might benefit the patients if recommended in clinical practice.

## Introduction

Neoadjuvant chemoradiotherapy and total mesorectal excision (TME) had dramatically reduced local recurrences and improved survival for patients with locally advanced mid-low rectal cancer 1, 2. Most studies in this field only focused on resectable rectal cancer 3-5, leaving the niche - the efficacy in unresectable rectal cancer - to be filled. The treatment for initially unresectable mid-low rectal cancer (IURC), which is commonly defined as a palpably fixed lesion involving adjacent structures or as a large nonmobile tumor 6, 7, is to achieve tumor shrinkage and R0 resection, i.e., conversion therapy. However, with limited data, optimal strategy and the efficacy of the conversion therapy for IURC patients remained undetermined.

Several studies have claimed that some chemoradiotherapy strategies have potential to improve the rate of pathologic complete response (pCR) and local control of mid-low rectal cancer. For examples, studies revealed that addition of fluorouracil and leucovorin to preoperative radiotherapy improved local control, the rate of R0 resection and cancer-specific survival compared with preoperative radiotherapy alone 5, 7. Also, CAO/ARO/AIO-94 study and FOWARC study indicated that adding oxaliplatin to neoadjuvant chemoradiotherapy significantly improved disease-free survival (DFS) and pCR rate with acceptable acute toxicity 8, 9. Besides, delayed surgery or several cycles of mFOLFOX6 between the end of chemoradiotherapy and TME could give rise to tumor downstaging 10, increase the rates of pCR and R0 resection and improve DFS 10-13.

Based on the previous studies, we have developed a conversion regimen for patients with IURC, which is concurrent administration of oxaliplatin and oral fluoropyrimidines during conventionally fractionated radiotherapy, followed by several cycles of consolidation chemotherapy and delayed surgery. In this study, we retrospectively assessed the efficacy and safety of this regimen in IURC patients and analyzed the prognosis, providing new insights into the management of IURC patients.

## Materials and Methods

### Patients

Medical records of patients with mid-low rectal cancer admitted to Zhongshan Hospital Fudan University from October 2010 to April 2017 have been reviewed. Patients with initially unresectable mid-low rectal cancer, which was defined as a primary tumor involving adjacent organs or structures (cT4bNxM0) or palpably fixed cT3N2 tumor that could not be radically resected (fixed cT3N2M0), were analyzed. Written informed consent was obtained from all patients before the start of the study and this study was approved by the Institutional Ethics Committee of Zhongshan Hospital Fudan University, and was conducted in accordance with the Declaration of Helsinki.

The inclusion criteria included histologically confirmed adenocarcinoma of the rectum, distance ≤10 centimeters from the anal verge to the inferior margin of the tumor determined by flexible colonoscopy, cT4b or fixed cT3N2 tumor without distant metastasis, and ASA score of 1-3. The exclusion criteria included history of previous malignant tumor, history of previous pelvic surgery, multiple colorectal cancers, severe diseases of other systems, and pregnant or breast-feeding women. The tumor height was measured by flexible colonoscopy, and the clinical stages of all patients were assessed by pelvic magnetic resonance imaging (MRI) and/or abdominopelvic computed tomography (CT).

### Conversion therapy regimen

All patients were evaluated by the multidisciplinary team (MDT) before receiving any therapy and were recommended to receive the conversion therapy containing three parts: 1) radiotherapy: a total of 45 Gy in 25 fractions (1.8 Gy per day, 5 days a week for 5 weeks), followed by a minimum boost of 5.4 Gy; 2) concurrent chemotherapy: oral capecitabine, 825 mg/m² twice a day from day 1 to 5 per week, and oxaliplatin, 50 mg/m² once per week at day 1, 8, 15, 22, 29 by continuous infusion throughout radiation; 3) delayed surgery and the consolidation chemotherapy: patients received 3 cycles of mFOLFOX6 or 2 cycles of Capox one week after the completion of radiotherapy. Each cycle of mFOLFOX6 consisted of leucovorin 400 mg/m², oxaliplatin 85 mg/m² in a 4-hour infusion, bolus fluorouracil 400 mg/m² on day 1, and a 48-hour infusion of fluorouracil 2400 mg/m². Each cycle of Capox consisted of capecitabine (1000 mg/m^2^ twice daily for 14 days every 3 weeks) and oxaliplatin (130 mg/m^2^ in a 4-hour infusion on day 1 of each cycle).

### Surgery and Pathologic Analysis

The second resectability evaluation by the MDT was performed approximately 8 weeks after completion of the chemoradiotherapy. Operation was recommended to the patients whose tumor shrank to be resectable and TME was performed approximately 2 weeks after the last cycle of consolidation chemotherapy. For those patients whose tumors were progressive or still remained unresected, further treatment was given according to the advises of the MDT. The type of surgery was determined according to the situation of intraoperative exploration on the premise of radical resection. Multiple organ resection was performed when the tumor was invasive to adjacent organs. Preventive ileostomy was performed in patients with high anastomotic tension.

Pathologic stages were recorded according to AJCC Cancer Staging Manual, 8th edition and distal margin involvement was documented. Pathological complete response was defined as ypT0N0M0, and pathologic downstaging was defined as lower pathologic T stage compared with the pretreatment clinical T stage. Tumor response to the conversion therapy was documented according the Modified Ryan classification recommended by NCCN Guidelines.

### Follow-up

All patients were routinely followed up every 3 to 6 months within the first two years, every half a year within 5 years and every year after 5 years. The examinations included serum carcinoembryonic antigen and abdominal ultrasonography at each time, CT scans every 6 to 12 months, and colonoscopy one year after operation and every 3 to 5 years. Local recurrence, distant metastasis and death event were documented once clinical or pathologic evidences were found.

### Statistics

Overall survival (OS) was calculated from the initiation of conversion therapy to death of any cause or the latest follow-up. Disease-free survival (DFS) was calculated from the date of R0 resection to recurrence, metastasis or the latest follow-up. Survival curves were estimated by the Kaplan-Meier method and compared using the log-rank test. *P* < 0.05 was considered to be statistically significant. All statistical evaluations were performed with SPSS software version 22.0.

## Results

### Patients

Sixty-one patients were included in the study. Patient baseline characteristics were showed in Table 1. The majority of the patients enrolled (77.0%) had cT4b primary tumors. All the cT3N2 tumors were palpably fixed and had presacral space lymph nodes involvement, and all the cT4b tumors had at least one adjacent organ involvement identified by at least one experienced radiologist, which made them unresectable (Table 1).

### Conversion therapy and toxicity events

The regimen and completion status of conversion therapy were presented in Table 2. In summary, 1) The established full dose of radiation was performed in all of the 61 patients; 2) 62.3% of the patients received concurrent chemotherapy with capecitabine plus oxaliplatin and 37.7% with capecitabine alone; 3) 46 (75.4%) patients received consolidation chemotherapy with mFOLFOX6 or Capox after the end of radiation, and 6 (9.8%) received capecitabine alone. The median number of cycles of consolidation chemotherapy was 2 (range from 1 to 3).

Acute adverse effects of the conversion therapy were showed in Table 3. Overall toxicity (grade 1-4) was observed in 29 (47.5%) patients, including that grade 1-2 toxicity occurred in 21 (34.4%) patients and grade 3 toxicity occurred in 8 (13.1%) patients. No grade 4 complication or treatment-related death has occurred. Bone marrow depression was the most common toxic effect occurred during the chemoradiotherapy, leading to drug withdrawal or drug reduces. Diarrhea was observed in 8 patients, and 2 of them reduced the dose of capecitabine.

### Response and surgery

At the second resectability evaluation, 54 (88.5%) patients had tumor size reduction, while 3 (4.9%) patients had stable-disease response, 3 (4.9%) had local progression and 1 (1.6%) had osseous metastasis. All of the 54 patients received surgery, and 51 (83.6%) of them received R0 resection, while 3 of them still had unresectable lesions determined during the operation and received alternative surgery, including 2 patients underwent sigmoidostomy and one received R2 resection. The median interval between completion of chemoradiotherapy and surgery was 64.5 days (IQR 57.5-72.5). After conversion therapy, a high response rate was observed in terms of the tumor regression grade (TRG) 0 to 1. Pathological complete response and downstaging were found in 10 (16.4%) and 38 (62.3%) patients, respectively. Details were shown in Table 4.

### Short-term outcomes and surgical complications

Operative details of patients who underwent R0 resection were shown in Table 5. Minimally invasive surgery was performed in 32 (62.7%) patients and the incidence of sphincter-sparing resection was 49%. The rate of combined organ resection, the operation time and the intraoperative blood loss were acceptable. Preventive ileostomy was performed in 3 patients. The postoperative acute adverse events were showed in Supplementary Table 1. These data suggested that the short-term outcomes were acceptable in the successful conversion patients.

### Long-term survival

**Figure 1:**
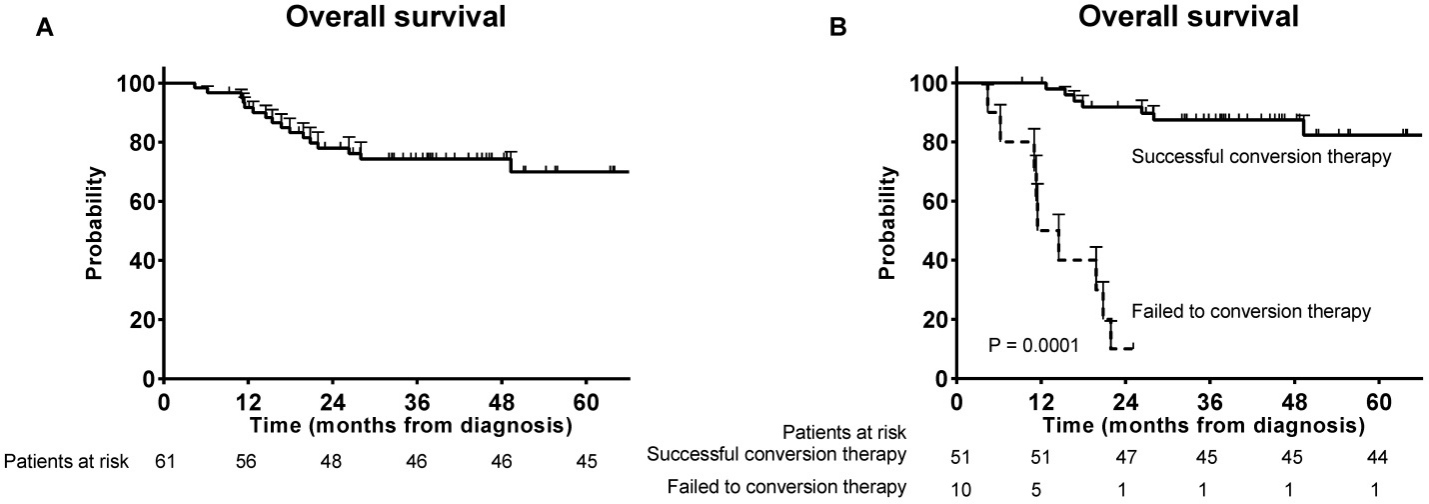
Overall survivals. (A) Overall survival for the patients who received conversion therapy; (B) Overall survival for the successful conversion patients and failed conversion patients.

The median follow-up time was 37.6 months overall. Seventeen patients died during follow-up and the OS rate at 3 years was 75.4% (Figure 1A). Significant survival benefit was observed in the successful conversion patients compared with those who failed to the conversion therapy (Figure 1B).

**Figure 2:**
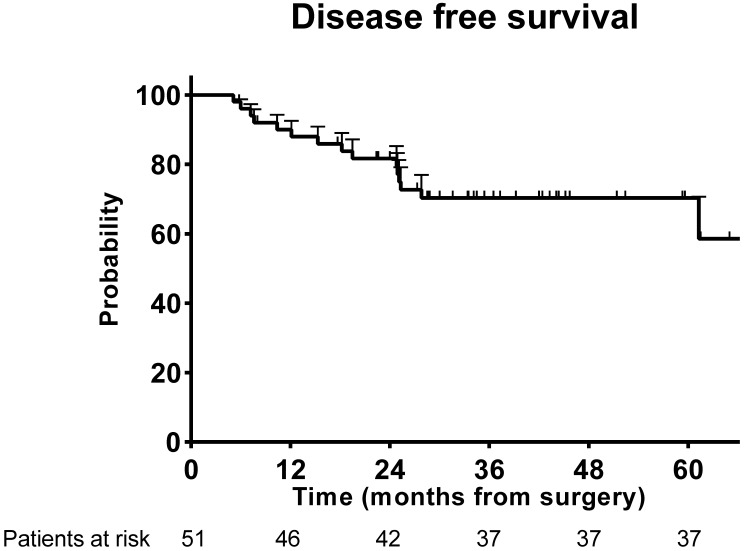
Disease-free survival for the successful conversion patients.

For patients who received R0 resection, total recurrence events occurred in 15 (29.4%) of the 51 patients. The DFS rate at 3 years was 72.5% in the successful conversion patients (Figure 2).

## Discussion

To our knowledge, the standard regimen for IURC patients and its outcomes still remained unclear. This study showed that patients with initially unresectable mid-low rectal cancer treated with this conversion therapy achieved a high rate of R0 resection with an desirable long-term survival and a low rate of grade 3-4 toxicity. This study could provide some reference for optimizing the management of IURC patients.

The rate of R0 resection in this study was higher than those in previous studies with similar population characteristics. Bujko and colleagues reported the rates to be 71%-77% in their study with two groups of conversion therapy 14, which specifically were 77% in the group treated with 5x5 Gy and three cycles of FOLFOX4 and 71% in the group treated with long-course simultaneously chemoradiotherapy. The better performance of our regimen could be attributed to the following strategies adopted in it. First, the delayed surgery was used in our regimen, which has been reported to be able to induce tumor downstaging 10-12, 15, 16. Second, our patients received consolidation chemotherapy during the interval of chemoradiotherapy and TME, which had been confirmed efficient in improving the pCR rate in previous studies 13, 17. Third, comparing to the former group which used short-course radiotherapy with 3 cycles of FOLFOX4, our R0 resection rate was slightly higher, probably because the concurrent chemotherapy may enhance the efficacy of preoperative treatment 18, even though no direct evidence was found to demonstrate the superiority of conventionally fractionated radiation to short-course radiation 16, 19. Finally, the addition of oxaliplatin to fluorouracil-based chemotherapy has been reported to be more efficient in preoperative treatment 8, 9.

Upon reviewing previous literatures for optimization of therapeutic strategies for nonresectable rectal cancer, we found that the successful conversion rate (83.6%) in our study was the same as that (83.6%) reported by a previous randomized controlled clinical trial 7, though our strategy was more aggressive. However, the inclusion criteria differed between the two studies, and the cases we were dealing with might be more challenging. In the previous studies, patients with fixed cT3 or cT4 lesions and recurrence tumor were enrolled, while in this study, the eligible patients had cT4b tumor or fixed cT3 tumor with lymph node metastases (fixed cT3N2) confirmed by baseline MRI and/or CT. Obviously, by adding lymph node metastasis (N stage) into cT3 tumor enrollment, the inclusion criteria in this study was stricter.

Comparing to previous studies, the long-term outcomes of this conversion therapy were desirable. The 3-year OS rate in unresectable rectal cancer patients reported in previous studies was 53% - 73% 7, 14. For another, the 3-year DFS rate reported in the FOWARC trial and the German CAO/ARO/AIO-04 study was 71.2% - 77.2% 8, 20. In this study, the 3-year OS rate and DFS rate were 75.4% and 72.5%, respectively, suggesting competitive efficacy of this conversion regimen.

The toxicity of this conversion regimen was acceptable. For one thing, the rate of chemoradiotherapy related grade 3-4 acute toxic effects (13.1%) was lower compared to the previously studies of preoperative chemoradiotherapy with oxaliplatin or consolidation chemotherapy (18%-40%) 3, 8, 9, 13, 21-23. The lower rate of toxicity could be attributed to the application of image-guided intensity-modulated radiotherapy which entailed higher target conformity and lower radiographic exposure of bowel and bladder 24, 25. For another, the postoperative complications occurred in 18 (35.2%) patients who achieved R0 resection, which was similar to the STAR-01 trial and the NSAPB R-04 trial 3, 23.

There are some limitations in this study. First, it was a retrospective study with a limited sample size. Second, 16.4% of the patients failed to obtain initial pelvic MRI at baseline due to implanted metal objects in patient's body or other reasons, and alternatively, contrast CT was performed and the results were evaluated by two experienced radiologists.

## Conclusion

In conclusion, this study proposed a conversion regimen which was proved safe and effective for IURC patients, contributing to high rate of R0 resection and superior survival outcomes. Although lacking clinical evidences of higher levels, this regimen definitely has promising performance on IURC treatment and may improve survival benefits.

## Supplementary Material

Supplementary table.

## Figures and Tables

**Table 1:** Baseline characteristics (N=61).

Characteristic	Number of patients n (%)
Sex	
Male	40 (65.6)
Female	21 (34.4)
Age (years)	
Mean (SD)	59.6 (13.4)
ASA score	
I	42 (68.9)
II	19 (31.1)
III	0 (0)
Distance between tumor and anal verge (cm)	
≤ 5	29 (47.5)
> 5 and ≤10	32 (52.5)
Clinical stages	
cT4bNanyM0 *	47 (77.0)
Sacrum	17 (27.9)
Prostate gland	10 (16.4)
Uterus	8 (13.1)
Seminal vesicle gland	7 (11.5)
Base of the bladder	7 (11.5)
Pelvic side wall/floor	6 (9.8)
Posterior wall of vagina	1 (1.6)
cT3N2M0	14 (23.0)
Lymph nodes in presacral space	14 (23.0)

Abbreviations: SD: standard deviation; ASA: American Society of Anesthesiologists; cm: centimeter. * Details of the involvement of adjacent organs or structures. Some tumors involving two or more organs or structures.

**Table 2:** Regimen of conversion therapy (N=61).

Characteristics	Number of patients n (%)
Radiotherapy	61 (100)
Concurrent chemotherapy regimen	
Capecitabine and oxaliplatin	38 (62.3)
Capecitabine alone	23 (37.7)
Concurrent chemotherapy with dose reduction*	7 (11.5)
Consolidation chemotherapy regimen	
mFOLFOX6	40 (65.6)
Capox	6 (9.8)
Capecitabine	6 (9.8)
None	9 (14.8)
Cycles of consolidation chemotherapy	
0	9 (14.8)
1	15 (24.6)
2	14 (23.0)
3	23 (37.7)

Abbreviations: mFOLFOX6: modified infusional fluorouracil, leucovorin, and oxaliplatin; Capox: capecitabine combined with oxaliplatin. * Dose reduction because acute toxicity.

**Table 3:** Complications related to conversion therapy (N=61).

Events *	All n (%)	Grade 1-2 n (%)	Grade3 n (%)
Toxicity (NCI-CTC version 3.0)	29 (47.5)	21 (34.4)	8 (13.1)
Hematological			
Leucopenia	10 (16.4)	8 (13.1)	2 (3.3)
Anemia	3 (4.9)	1 (1.6)	2 (3.3)
Infection or fever	3 (4.9)	2 (3.3)	1 (1.6)
Gastrointestinal			
Diarrhea	8 (13.1)	5 (8.2)	3 (4.9)
Nausea or vomiting	2 (3.3)	1 (1.6)	1 (1.6)
Radiation procitis	9 (14.8)	8 (13.1)	1 (1.6)
Hand-foot syndrome	4 (6.6)	3 (4.9)	1 (1.6)
Radiation dermatitis	8 (13.1)	7 (11.5)	1 (1.6)

Abbreviation: NCI-CTC: National Cancer Institute-Common Toxicity Criteria. * Some patients experienced more than one toxicity event; therefore, the totals may exceed 100%.

**Table 4:** Response of conversion therapy (N=61).

Characteristics	Number of patients n (%)
Clinical evaluation	
Clinical downstaging	54 (88.5)
Stable disease	3 (4.9)
Local progression	3 (4.9)
Distant metastases	1 (1.6)
Received surgery	
No	7 (11.5)
Yes *	54 (88.5)
< 8w	9 (14.8)
≥ 8w; <12w	37 (60.7)
≥ 12w	8 (13.1)
Conversion results #	
Successful	51 (83.6)
Fail	10 (16.4)
No surgery	7 (11.5)
R2 resection	1 (1.6)
Sigmoidostomy	2 (3.3)
Tumor regression grade	
0	10 (16.4)
1	21 (34.4)
2	5 (8.2)
3	15 (24.6)
pCR	10 (16.4)
Pathological downstaging	38 (62.3)

Abbreviations: RT: radiotherapy; w: week; pCR: pathological complete response. * Interval between the completion of chemoradiotherapy and TME for patients who received surgery # Successful conversion was defined as R0 resection after conversion therapy. Fail conversion was defined as still unresectable tumor after conversion therapy.

**Table 5:** Short-term outcomes for the successful conversion patients (N=51).

Characteristics	Successful conversion n (%)
Surgical procedures	
Abdominoperineal resection	26 (51.0)
Low anterior resection	23 (45.1)
Hartmann	2 (3.9)
Minimally invasive surgery	
Yes	32 (62.7)
No	19 (37.3)
Protective ileostomy	3/23 (13.0)
Combined organ resection	4 (7.8)
Operative time (min)	162.5 ± 22.7
Intraoperative blood loss (ml)	74 ± 59
Postoperative complications	18 (35.2)
Reoperations	0 (0)
Hospitalized days after surgery, median (range)	7 (4-28)
Urinary catheter, (day)	4.1 ± 3.9
Liquid diet, (day)	3.3 ± 2.5
