# Supplementary material for: Efficacy of conversion therapy on initially unresectable locally advanced rectal cancer
Source: J Cancer. 2021 May 27;12(14):4418–23. doi: 10.7150/jca.53824 (PMC8176423; doi:10.7150/jca.53824)
Supplement: Supplementary file 1 — Supplementary table. [file jcav12p4418s1.pdf]

Table S1. Postoperative complications of patients who received R0 resection (N = 51).

**Characteristics**	**Number of patients n (%)**
Postoperative complications	18 (35.3)
Grade 3 anastomotic leakage	0/23 (0)
Surgical site infection (SSI)	3 (5.9)
Chyle leak	1 (2.0)
Gastrointestinal motility disorders	2 (3.9)
Abdominal infection	6 (11.8)
Urinary retention	5 (9.8)
Hematochezia	1 (2.0)
Urinary tract infection	1 (2.0)
Others	0 (0)
